# MANAGEMENT AND FOLLOW-UP OF EXTENSIVE TERATOID CYST IN MOUTH FLOOR

**DOI:** 10.1590/0102-6720201600020015

**Published:** 2016

**Authors:** Emeline das Neves DE ARAÚJO LIMA, Márcio Menezes NOVAES, Adriano Rocha GERMANO, José Sandro Pereira da SILVA, Lélia Batista de SOUZA

**Affiliations:** Universidade Federal do Rio Grande do Norte (Federal University of Rio Grande do Norte), Natal, RN, Brazil.

**Keywords:** Teratoma, Dermoid cyst, Mouth floor, Child

## INTRODUCTION

Dysontogenetic cysts, commonly referred as dermoid cysts or teratoid cysts, are hamartomas which may contain various derivatives of endoderm, mesoderm and ectoderm^7^. The majority of cases is reported in the midline of the body and especially in testes and ovaries. The most common site in the head and neck region is the lateral eyebrow, the so-called angular dermoid, and approximately 6.5% of the cases occur in the oral cavity. The teratoid cyst of the floor of the mouth is distinctly uncommon, with only a few cases reported, usually in the anterior portion^8^ ^,^ ^14^.

Three theories with regard to the origin of cysts in the floor of the mouth were found in literature. According to the 1^st^ and most prevalent theory, these cysts originate from embryonic cells of the 1^st^ and 2^nd^ branchial arches during the 3^rd^/4^th^ week of embryonic life. The 2^nd^ theory explains the pathogenic mechanism of the acquired form, which may be due to the implantation of epithelial cells subsequent to accidental or surgical injury (traumatic causes, iatrogenic antecedents, or an occlusion of a sebaceous gland duct). Lastly, the 3^rd^ theory maintains that these cysts are considered a variation of the cyst of the thyroglossal pore^6^. With regard to the etiology of dermoid and teratoid cysts in this site, there is much theory, but the most accepted is a possible sequestration of ectodermal tissue in the midline at the time of fusion of the first (mandibular) and second (hyoid) brachial arches^2^ ^,^ ^10^.

Histologically, the dermoid cyst differs from epidermoid cyst only in the presence of normal or dysmorphic adnexal appendages within its walls, usually sebaceous glands or abortive hair follicles. The teratoid cyst is considered if the cyst wall contains other elements, such as muscle or bone^11^. Surgical approaches for excision have been the treatment of choice for dermoid or teratoid cyst, including intraoral and extraoral skin incisions^12^. Most of the authors recommend conservative surgical removal, trying not to rupture the cyst, as the luminal contents may act as irritants to fibrovascular tissues, producing postoperative inflammation. Recurrence and malignant transformation of oral cysts are unlikely after treatment^8^ ^,^ ^9^.

This paper presents a case of teratoid cyst in a child with emphasis on the management and follow-up of six months.

## CASE REPORT

**FIGURE 1:**
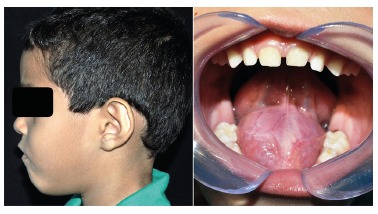
Extraoral examination showing volume increase in the submental region and intraoral examination showing nodular mass causing proptosis of the tongue

**FIGURE 2:**
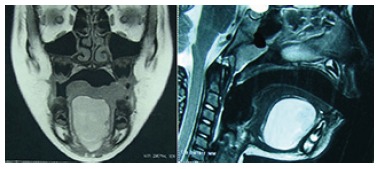
Magnetic resonance imaging showing oval cystic formation and rounded images with intermediate signal intensity on T1 and T2 weighted sequences

**FIGURE 3:**
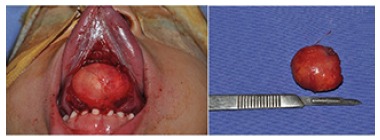
Surgical enucleation after blunt dissection of the well-encapsulated mass

**FIGURE 4:**
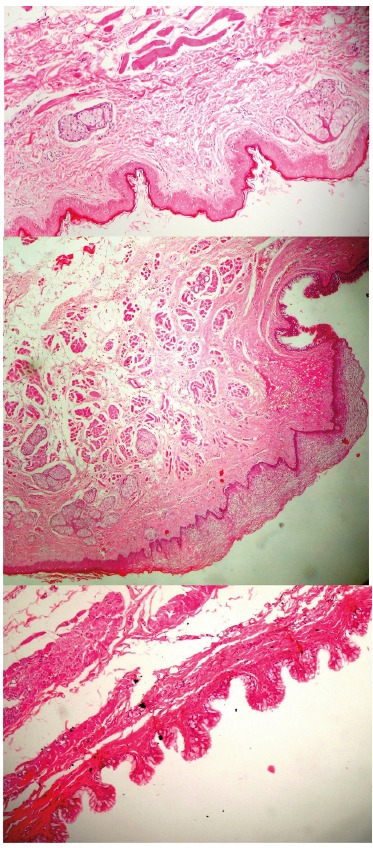
Photomicrography showing the orthokeratotic stratified squamous epithelium, the gastrointestinal epithelium and the transitional parakeratotic stratified squamous epithelium (H/E stain, magnification × 40 and details in H/E stain, magnification × 100)

**FIGURE 5:**
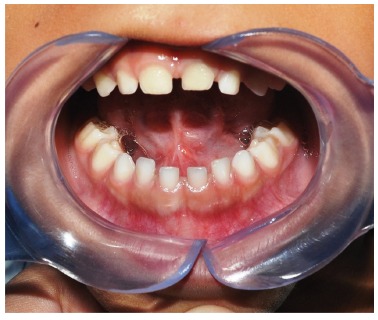
Follow-up of the patient six months after the surgery

5-year-old male attended the oral diagnostic service, reporting swelling in the mouth floor, with time course of approximately three months. In extraoral examination there was evidence of a slight volume increase in the submental region of about 3 cm with floating consistency. The intraoral examination showed proptosis of the tongue with no change in the overlying mucosa (Figure 1). Magnetic resonance imaging showed an oval cystic formation, measuring 2.6x4.5x3.1 cm, located on the floor of the mouth, without evidence of bone erosion or infiltration of adjacent muscle. Resonance also showed small rounded images with intermediate signal intensity on T1 and T2 weighted sequences (Figure 2). The clinical diagnosis was dermoid cyst and the tumor was excised by blunt dissection until the complete removal without any rupture of the cystic capsule (Figure 3). Microscopic analysis revealed a dermoid cyst associated with oral heterotopic gastrointestinal cyst, characterized by a cavity lined by orthokeratotic stratified squamous epithelium, with areas of gastrointestinal epithelium showing microvilli and the presence of goblet cells. It was also noted the presence of hair follicles and sebaceous glands in the capsule underlying the orthokeratotic epithelium. In some areas, it was possible to see the transition between the orthokeratotic and the gastrointestinal epithelium and, at this point, it was observed the presence of parakeratotic stratified squamous epithelium (Figure 4). The histopathologic findings were consistent with those of a mature teratoid cyst and the patient showed no clinical signs of recurrence six months after surgical excision (Figure 5).

## DISCUSSION

During the formation of the face and neck, branchial arches fuse in the midline between the third and fourth weeks of intrauterine life. It is believed that congenital dermoid cysts are a result of entrapment of a fragment of ectodermal tissue in the midline, just behind the mandible. Some of these trapped cells are totipotential blastomeres that can develop into any of the three germ layers^1^. Acquired dermoid cysts arise from epithelium implanted during trauma, and they often occur at sites away from the midline^8^. The term "teratoid cyst" was first used by Meyer^13^ in his classification of dysontogenetic cysts of the cervicofacial region, based on the type of the germinative layers included in the cystic wall. He defined three distinct histological types: epidermoid (simple), dermoid (compound) and teratoid (complex). An epidermoid cyst is always lined by stratified squamous epithelium without dermal appendages within the underlying connective tissue. Dermoid cyst, in addition to the typical squamous epithelium, contains dermal appendages, such as hair, hair follicles, sebaceous and sweat glands. The wall of the teratoid cyst is also lined with squamous epithelium, but it can consist of tissues from all three germ layers, such as those of the respiratory, gastrointestinal and nervous system. The lumen of all three types of cysts displays a greasy, cheese-like, white-gray or yellow-tan content, formed by shed keratin and sebaceous material^10^ ^,^ ^13^.

Dermoid and teratoid cysts represent approximately 1.8% of all mouth floor cysts, and such lesions are very rare in infancy^3^ ^,^ ^5^ ^,^ ^10^, differently of the case reported. These cysts can be misdiagnosed with a large number of diseases which occur in this area with similar clinical aspects and symptomatology^4^. Differential diagnosis should include developmental lesions, congenital, inflammatory and salivary gland lesions, lymphomas and benign tumors^6^. The differential diagnosis of teratoid cyst in the floor of the mouth includes thyroglossal duct cyst, ranula, cystic hygroma, branchial cleft cysts, benign mesenchymal tumors, benign and malignant salivary gland tumors, Hodgkin's disease and non-Hodgkin's lymphoma and infections^7^ ^,^ ^9^. The precise diagnosis of these diseases can be made after an appropriate clinical examination and imaging investigation^11^ ^,^ ^14^. When lined by squamous cells, differentiation between a thyroglossal duct cyst and teratoid cyst can be difficult^4^.

The treatment of choice for cysts in the floor of the mouth is their total extraction (enucleation) via intraoral or extraoral approach or a combination of both, determined on each occasion by the location and size of the cyst^4^. In most cases, the enucleation can be carried out intraorally, as clearly evident in a review of international bibliography, which found that in 120 cysts surgically treated, 70 (58%) were done intraorally, 37 (31%) extraorally, and 13 (11%) via a combination of intra and extraoral approaches^6^. Effective treatment of dermoid and teratoid cyst of the floor of the mouth requires identification and surgical excision of any tracts leading to the midline of the mandible or hyoid bone. Failure to eliminate these epithelium-lined structures is stated to increase risk of recurrence^14^ ^,^ ^15^. The cyst described here was completely excised by intraoral approach, which was determined by the location of the cysts on higher planes. A broad surgical field was obtained, allowing a blunt dissection and full removal of the cyst, without break of the capsule, reducing the chances of recurrence. On histopathological examination, the presence of gastrointestinal epithelium, along with hair follicles and sebaceous glands in the capsule confirmed the final diagnosis of a teratoid cyst.
